# Effect of hip bracing on stair walking biomechanics and pain in patients with mild-to-moderate hip osteoarthritis: an intervention study

**DOI:** 10.1186/s12891-026-09587-2

**Published:** 2026-02-11

**Authors:** Hannah Steingrebe, Hannah Ehmann, Stefan Sell, Thorsten Stein

**Affiliations:** 1 BioMotion Center, Institute of Sports and Sports Science, Karlsruhe Institute of Technology (KIT), Engler-Bunte-Ring 15, Karlsruhe, 76131 Germany; 2 Sports Orthopedics, Institute of Sports and Sports Science, Karlsruhe Institute of Technology (KIT), Karlsruhe, Germany; 3 Joint Center Black Forest, Hospital Neuenbürg, Neuenbürg, Germany

**Keywords:** Hip osteoarthritis, Conservative management, Stair negotiation, Orthoses, Kinematics, Pelvis motion, Hip moments, Bracing, Pain

## Abstract

**Background:**

Bracing is a conservative treatment method for hip osteoarthritis (HOA) and has shown favourable effects on pain and functional capacity. However, biomechanical analyses of brace effects remain sparse and are limited to level walking. Stair walking is more demanding than level walking in terms of movement coordination and joint loads. This study, therefore, aimed to investigate the effect of hip bracing on pain perception and biomechanics of the hip, pelvis, and trunk during stair walking in individuals with HOA.

**Methods:**

Hip, pelvis, and trunk biomechanics and pain during stair ascent and descent were assessed before and after one week of hip bracing in 20 individuals with unilateral mild-to-moderate HOA. Differences between the bracing conditions were analysed with dependent t-tests, and Pearson’s correlations were used to analyse the correlation between brace-induced alterations in pain score and biomechanical parameters.

**Results:**

Bracing increased movement velocity and reduced stair walking pain by 28%. Furthermore, increased hip extension and reduced hip flexion were found with bracing. Bracing led to a decrease in anterior pelvis tilt, resulting in a more upright pelvis position. Trunk motion was not affected by bracing. During stair ascent, frontal pelvis motion increased, while peak hip adduction and internal rotation decreased with bracing. During stair descent, increased hip extension and external rotation moments were found with bracing, while the pelvis and hip transverse range of motion were reduced. Decreased pelvis rise on the ipsilateral side during stair ascent and increased hip transverse range of motion during stair descent were moderately correlated with a decrease in pain.

**Conclusions:**

Bracing can reduce hip pain during stair walking and mitigate some of the effects of HOA on stair walking biomechanics, making it a valuable conservative treatment option for individuals with mild-to-moderate HOA. Limiting hip internal rotation exclusively during periods of high joint loading could be a promising mechanism for reducing pain in individuals with HOA. The observed biomechanical changes are indicative of altered hip abductor muscle activity and increased joint loading. Hence, further analyses are necessary to explore the relationship between hip bracing, muscle activity, joint loading and pain.

**Supplementary Information:**

The online version contains supplementary material available at 10.1186/s12891-026-09587-2.

## Background

Hip osteoarthritis (HOA) is a chronic, progressive disease whose prevalence increases with age [1]. Despite its widespread occurrence, recommendations for HOA management are often extrapolated from knee osteoarthritis (KOA) research [2, 3]. Furthermore, the majority of HOA research has concentrated on addressing end-stage HOA. By shifting the focus of disease management to the earlier stages of the disease, it may be possible to slow disease progression, enhance patient quality of life, and ultimately reduce the number of hip replacement surgeries [3]. Conservative management of HOA aims to reduce joint pain and stiffness, maintain or improve joint mobility and stability, enhance activities and participation, and improve the quality of life [2, 4]. Thereby, it has been shown that individuals with HOA have a strong desire to self-manage their condition [5].

One option for conservative HOA management that can easily be regulated by the patient is hip bracing. Hip bracing for HOA has shown favourable effects on pain and functional capacity [6–8]. However, biomechanical analyses of bracing effects in HOA are sparse and limited to level walking movements [7, 9]. Prior biomechanical studies have related hip pain in HOA to joint compression due to hip abductor muscle activity and reaching of range of motion (RoM) endpoints, especially in hip extension and internal rotation [10–12]. A hip brace aiming to decrease load at the hip joint was able to reduce the peak hip adduction and internal rotation angles as well as the peak hip abduction moment, however with mixed effects on pain [9]. Using an elastic hip brace, which targets the soft tissue surrounding the hip and pelvis, resulted in reduced pain in most individuals and increased gait speed, step length, and peak hip extension moments during level walking [7].

Although walking on level ground is one of the most fundamental daily movements, walking up and down stairs is more demanding due to the greater sagittal and frontal joint angles of the lower limbs, greater moments at the hip and knee joints [13–15], and greater hip joint contact forces [16]. This high demand leads to lower performance during stair walking tests of individuals with various stages of HOA severity compared to healthy controls [17–19]. Furthermore, stair walking ability decreased with HOA progression [17, 20].

Stair walking in individuals with various degrees of HOA differs from healthy controls in terms of lower movement velocity, increased trunk flexion towards the affected limb and lower hip RoM in all planes, caused by lower hip flexion, extension, abduction, adduction and external rotation angles [10, 21–23]. Additionally, in one study, increased hip internal rotation angles were found [22]. Furthermore, hip extension, adduction and internal and external rotation moments [10, 21], as well as peak hip contact forces [24], are lower in individuals with HOA.

Thus, stair walking motion is impaired at all levels of HOA. However, to date, it remains unclear whether hip bracing can influence stair walking biomechanics and enhance stair walking ability. Therefore, the primary objective of the present study was to evaluate the impact of bracing on hip pain and biomechanics of the hip, pelvis, and trunk during stair walking in individuals with mild-to-moderate HOA. As the elastic brace used in this study intends to reduce pain, improve joint mobility and normalize biomechanical stair walking patterns, we expected bracing to reduce hip pain, peak hip internal rotation angles and ipsilateral trunk lean and increase movement velocity, frontal hip mobility and hip internal rotation moments during stair walking. The second aim of this study was to explore which brace-induced biomechanical changes were associated with changes in pain perception during stair walking.

## Methods

Data for this intervention study were collected between April 2019 and March 2021 in the BioMotion Center of the Institute of Sports and Sports Science at the Karlsruhe Institute of Technology.

### Participants

The cohort for this study consisted of 21 individuals (10 females; age: 64.0 ± 9.6 years; BMI: 24.2 ± 2.9) with symptomatic, functionally mild-to-moderate unilateral HOA. Participants had radiographically confirmed HOA of Kellgren-Lawrence (K-L) grade 2–4 (K-L 2 = 9 subjects, K-L 3 = 8 subjects, K-L 4 = 4 subjects) and experienced hip pain during daily activities over the past three months. Due to the weak association between radiographic HOA features and functional impairments [25], mild-to-moderate symptoms were defined functionally by a Harris Hip Score ranging from 65 to 95 (74.6 ± 11.8). None of the participants in our study reported experiencing extreme pain while walking up or down stairs as measured by the Hip Osteoarthritis Outcome Score [26]. Additionally, all participants were able to ascend and descend stairs in a step-over manner without using the handrail.

The contralateral limb needed to be pain-free, possess a K-L grade ≤ 2, and demonstrate an unrestricted passive joint RoM (sagittal RoM ≥ 90°, transverse RoM ≥ 15°, peak abduction ≥ 20°, flexing contracture ≤ 10°). Exclusion criteria included secondary HOA resulting from trauma, neuromuscular disorders or neurological complaints, a BMI of ≥ 35 kg/m^2^, or other orthopaedic injuries of the lower limbs and back.

The study was conducted in accordance with the guidelines of the Declaration of Helsinki and was approved by the Ethics Committee of the Karlsruhe Institute of Technology. All participants provided written informed consent prior to participating in the study.

### Hip brace

**Fig. 1:**
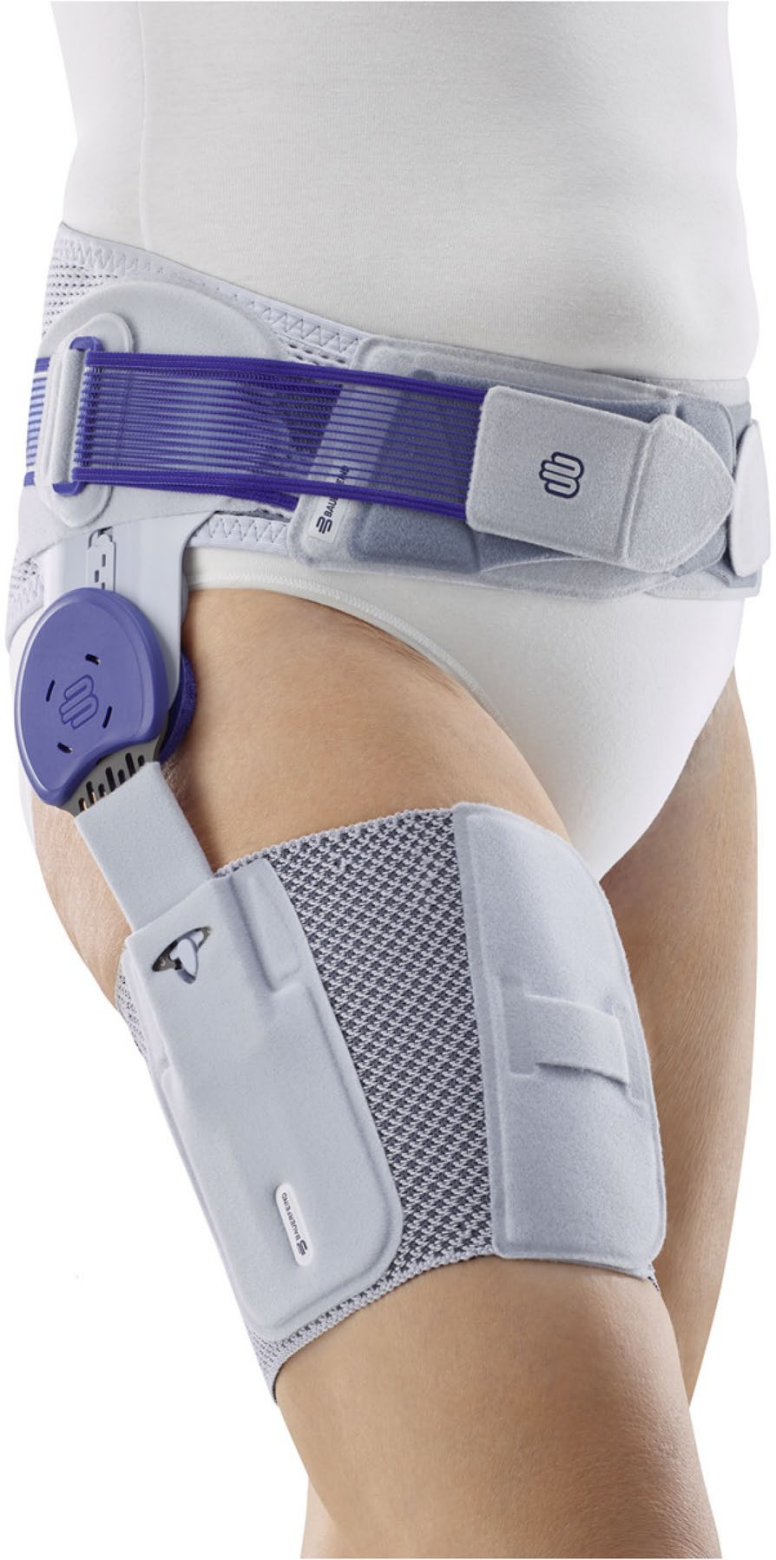
CoxaTrain (Bauerfeind AG) hip brace. ©Bauerfeind AG

The hip brace used in this study (CoxaTrain®, Bauerfeind AG; Fig. 1) comprises three components: an elastic pelvis belt, an elastic thigh belt, and a rigid connecting piece featuring a hinge joint that permits one degree of freedom (flexion/extension). Within the pelvis belt, viscoelastic pads are situated around the sacroiliac joint (SIJ) and the gluteus medius (GMed) regions. The rigid connecting piece includes a fixed pad at the greater trochanter and a movable pad positioned above it. The movable pad shifts vertically as the hinge joint flexes and extends. The purpose of the brace is to stabilise the pelvis girdle and SIJ, alleviate pain through trigger point massage of the SIJ and GMed regions, and restore functional hip mobility through continuous friction of GMed.

### Testing protocol

Participants visited the lab three times: on the first occasion, baseline gait analysis was conducted without bracing. Subsequently, participants recorded their pain perception during stair walking on a 10 cm visual analogue scale (VAS; anchored: no pain/worst imaginable pain) once a day for one week (baseline period). During the second visit, participants were individually fitted with a hip brace. Thereafter, participants wore the brace for one week (prescribed wear time > 4 h per day) during everyday activities and simultaneously recorded their pain perception during stair walking (braced period). During the third visit, gait analysis was repeated while wearing the brace (braced). In each analysis, participants performed five valid trials (no handrail use, no tripping) of stair ascent and descent at a self-selected speed in a step-over manner. Stair ascent was initiated one or two steps in front of the staircase at ground level, while stair descent began without approach steps from the top platform of the staircase. VAS pain scores were averaged across the seven days of each period.

### Data acquisition, data processing and biomechanical modelling

**Fig. 2:**
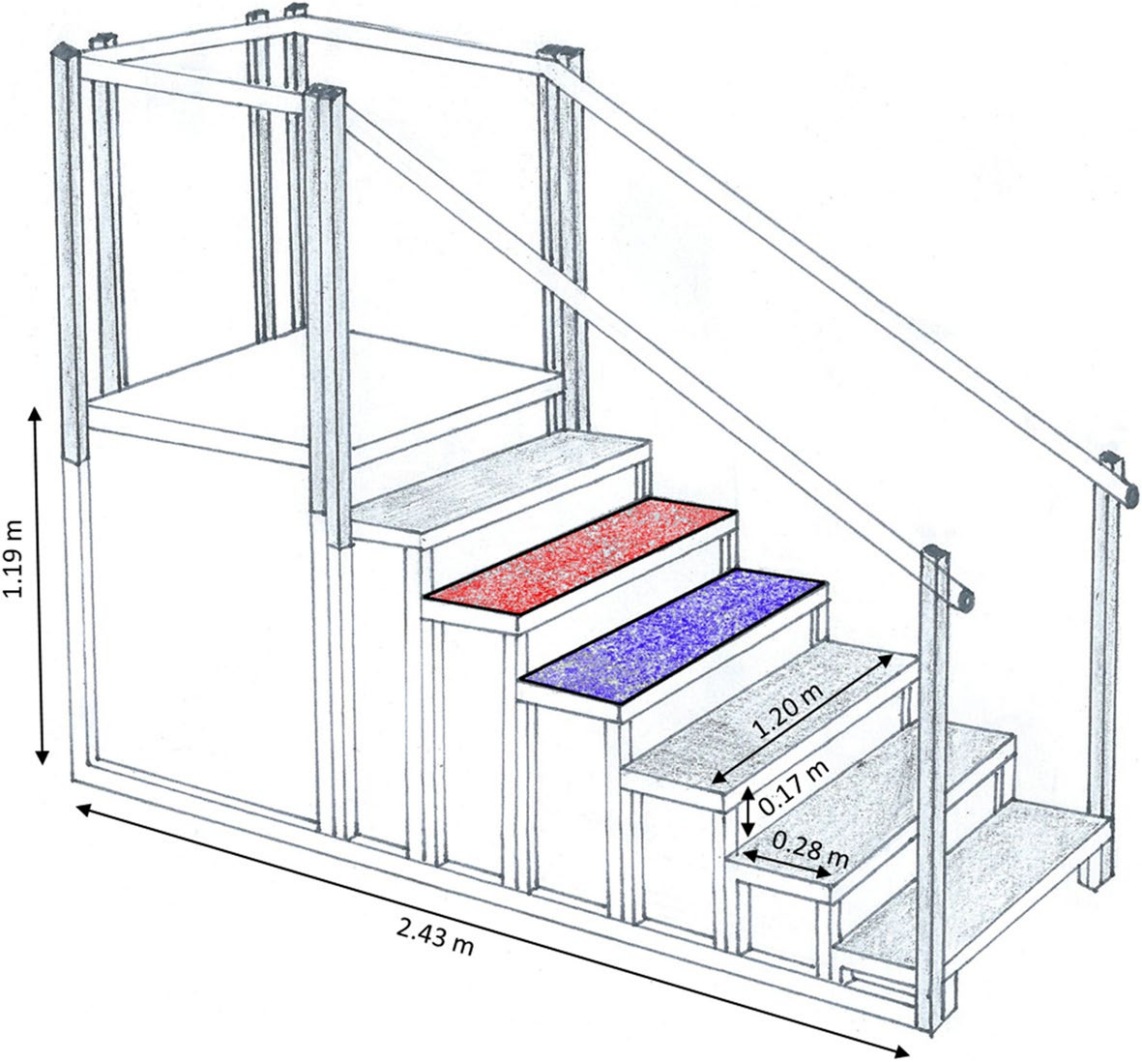
Sketch of the instrumented staircase. Three-dimensional force plates in steps 4 (blue) and 5 (red). Riser height = 17 cm, tread = 28 cm, inclination angle = 31.26°

Hip, pelvis, and trunk biomechanics were assessed using a 16-camera infrared motion capture system (200 Hz; Vicon Motion Systems, Oxford Metrics Group, Oxford, UK) and a custom-built instrumented staircase with six steps (riser height = 17 cm, tread width = 28 cm, inclination angle = 31.26°). Steps one to three and six were instrumented with 1D strain gauges for segmentation purposes (1000 Hz, Wii Balance Board, Nintendo, Kyoto, Japan), while steps four and five were equipped with 3D strain gauges (1000 Hz; K3D120 ± 1kN; ME-Meßsysteme GmbH, Henningsdorf, Germany) (Fig. 2). Participants were equipped with a full-body marker set consisting of 42 retroreflective markers, and 65 anthropometric measures were taken for individual scaling of the biomechanical model [27]. In the braced condition, the anterior superior iliac spines were covered by the brace, necessitating the application of markers on the brace. The markers on the posterior superior iliac spines were affixed through the mesh fabric of the brace.

Postprocessing of the data was conducted in Vicon Nexus (version 2.14.0) and MATLAB (version R2022a; The MathWorks Inc., Natick, MA, USA). Kinematic and ground reaction force (GRF) data were filtered using a 4th-order Butterworth low-pass filter with a cut-off frequency of 10 Hz [28]. 3D hip joint angles and external joint moments, as well as pelvis orientation, were calculated using an inverse kinematics and dynamics approach with the multi-body model ALASKA Dynamicus [27], including the hip joint centre definition proposed by Harrington [29]. Hip joint moments were normalised to body mass. Additionally, sagittal and frontal trunk angles were determined as the angle formed between a vector extending from the midpoint of both anterior superior iliac spine markers to the clavicle marker and the vertical axis [23]. The evaluated gait cycle started with initial contact (IC) on the 4th step and ended with IC on the 6th step for stair ascent, or started with IC on the 5th step and ended with IC on the 3rd step for descent. IC was detected by a vertical GRF > 15 N. The dependent variables were peak hip joint angles, hip RoM and peak hip joint moments, peak pelvis orientation angles and RoM, peak sagittal and frontal trunk angles, mean vertical and horizontal centre of mass (CoM) velocity, gait cycle duration, and stance phase duration.

Brace wear time was assessed using temperature sensors (Orthotimer®, Rollerwerk Medical Engineering and Consulting, Balingen, Germany) on the inside of the pelvis belt. Sensor data were cross-checked with a wear diary self-recorded by the participants.

### Statistical analysis

The required sample size was estimated through a priori power analysis using G*Power (version 3.1.9.3; [30]), based on data from Nérot and Nicholls [9]. They observed a bracing effect of 0.92 and 1.17 during walking for peak hip adduction and internal rotation, respectively. Consequently, with α = 0.05 and power = 0.95, the minimum sample size necessary was between 12 and 18 subjects.

Statistical analyses were performed using R (version 4.2.2; [31]). The normal distribution of residuals was assessed with the Shapiro–Wilk test. Effects were analysed using t-tests for dependent samples or the Wilcoxon signed rank test when the normal distribution of residuals could not be assumed. The level of significance was set a priori to ɑ < 0.05. Cohen’s d was used to estimate the effect size and was interpreted as |d |≥ 0.2 a small effect, |d| ≥ 0.5 a moderate effect and |d |≥ 0.8 a large effect [32].

To evaluate the relationship between changes in pain perception and biomechanical variables, Pearson’s correlations were calculated between the change in pain score and the change in biomechanical variables significantly affected by brace application. Correlation coefficients were interpreted as |r| ≥ 0.1 a small effect, |r| ≥ 0.3 a moderate effect and |r| ≥ 0.5 a large effect [32].

Due to a technical error, the GRF data for one of the 21 participants were not recorded during the third session. Consequently, all analyses were conducted using the data from the remaining 20 participants.

## Results

**Fig. 3:**
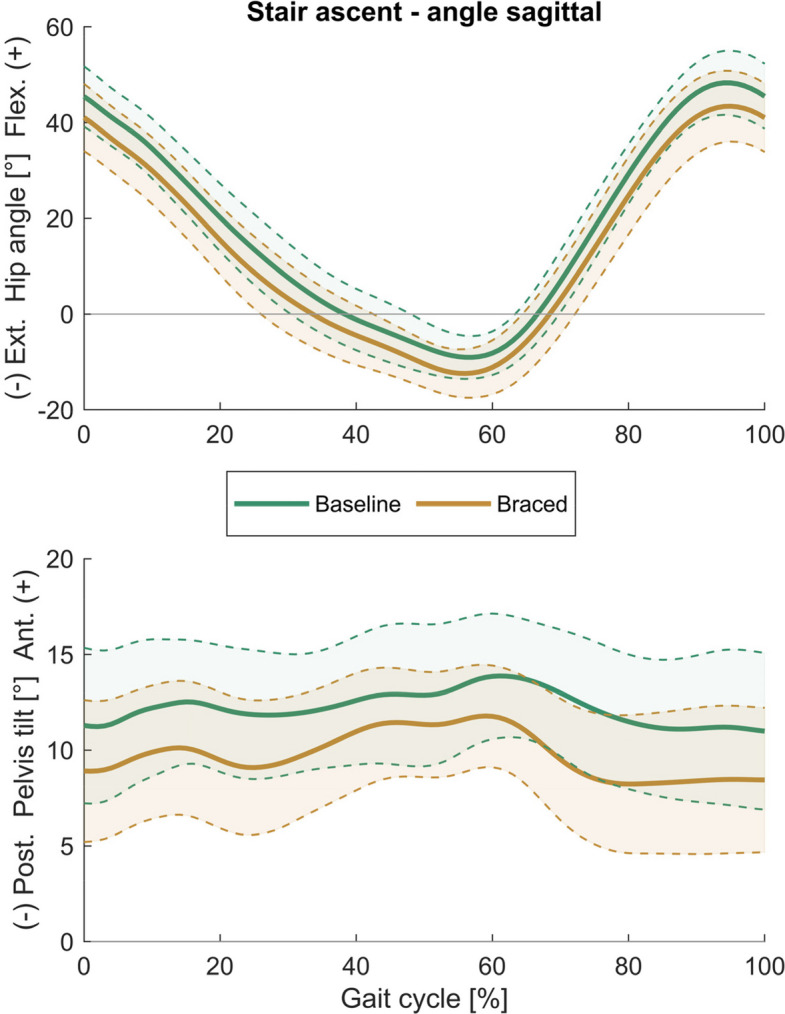
Sagittal plane hip and pelvis angles during stair ascent. (Mean ± SD)

**Fig. 4:**
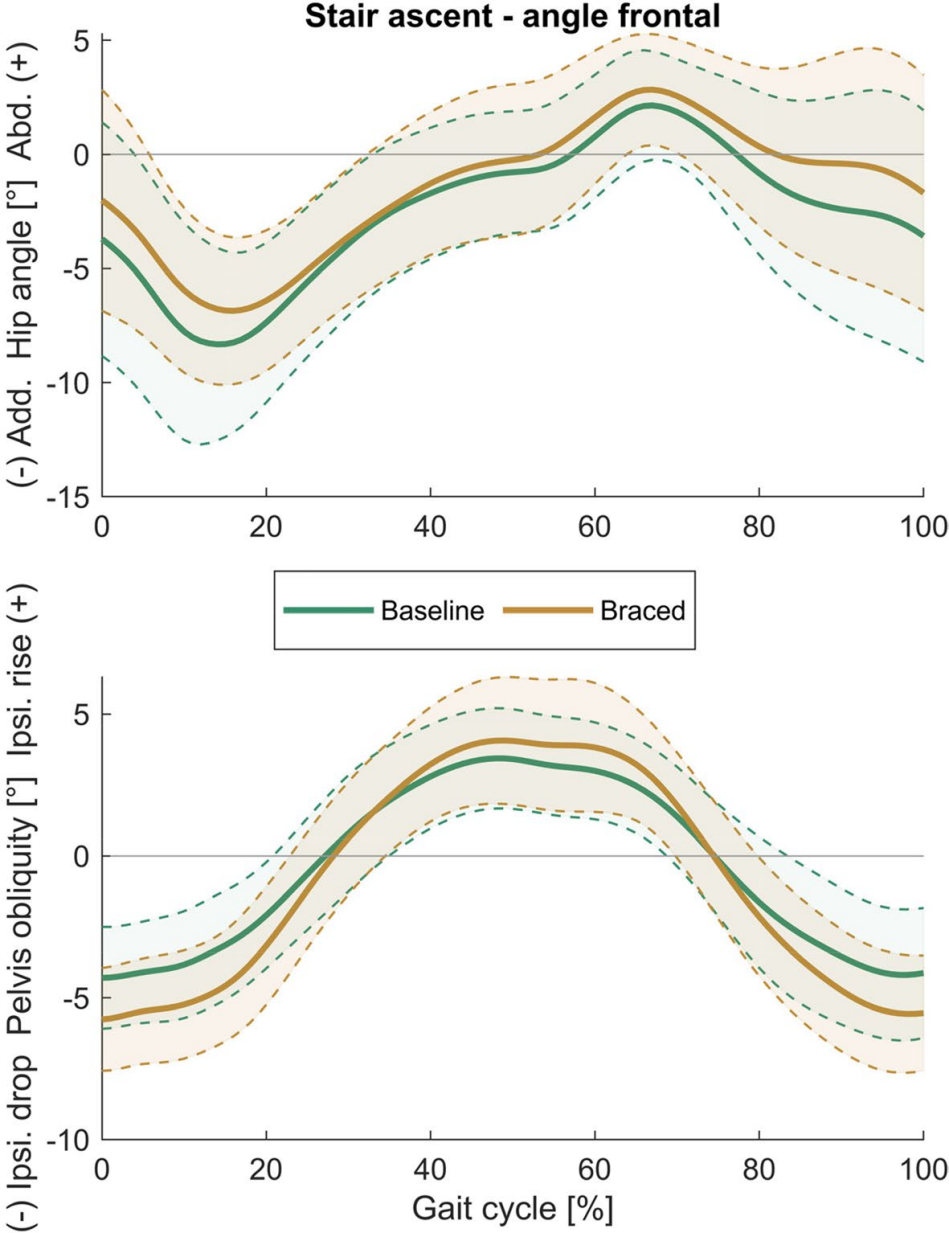
Frontal plane hip and pelvis angles during stair ascent. (Mean ± SD)

**Fig. 5:**
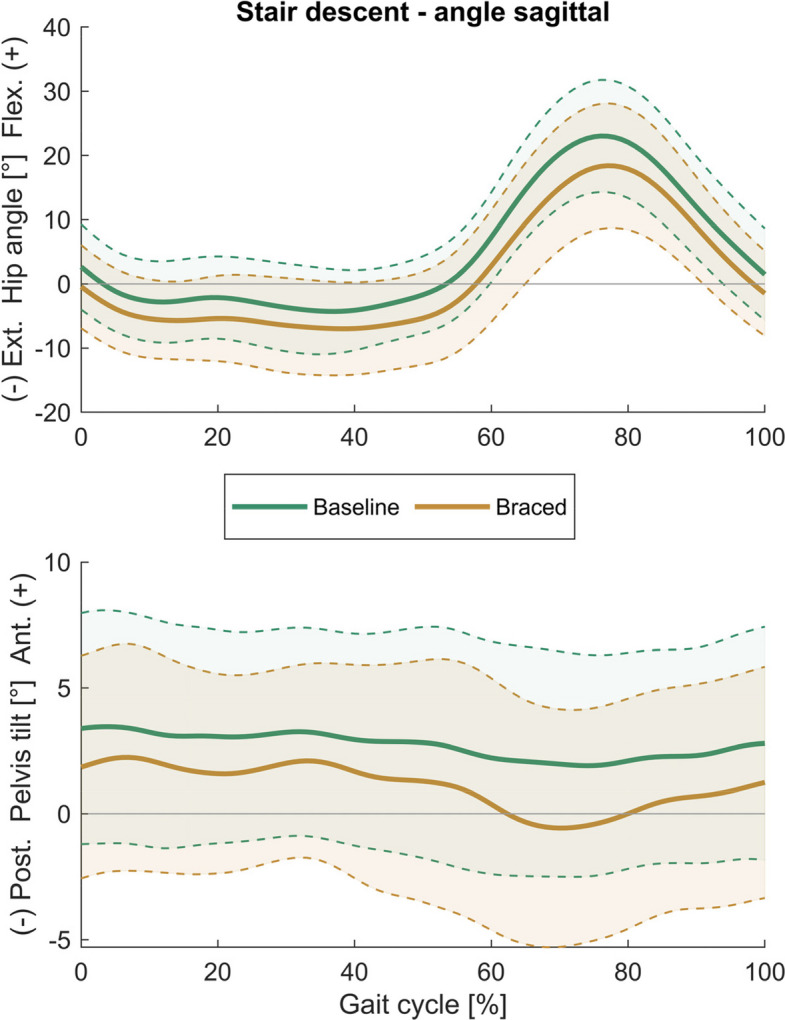
Sagittal plane hip and pelvis angles during stair descent. (Mean ± SD)

**Fig. 6:**
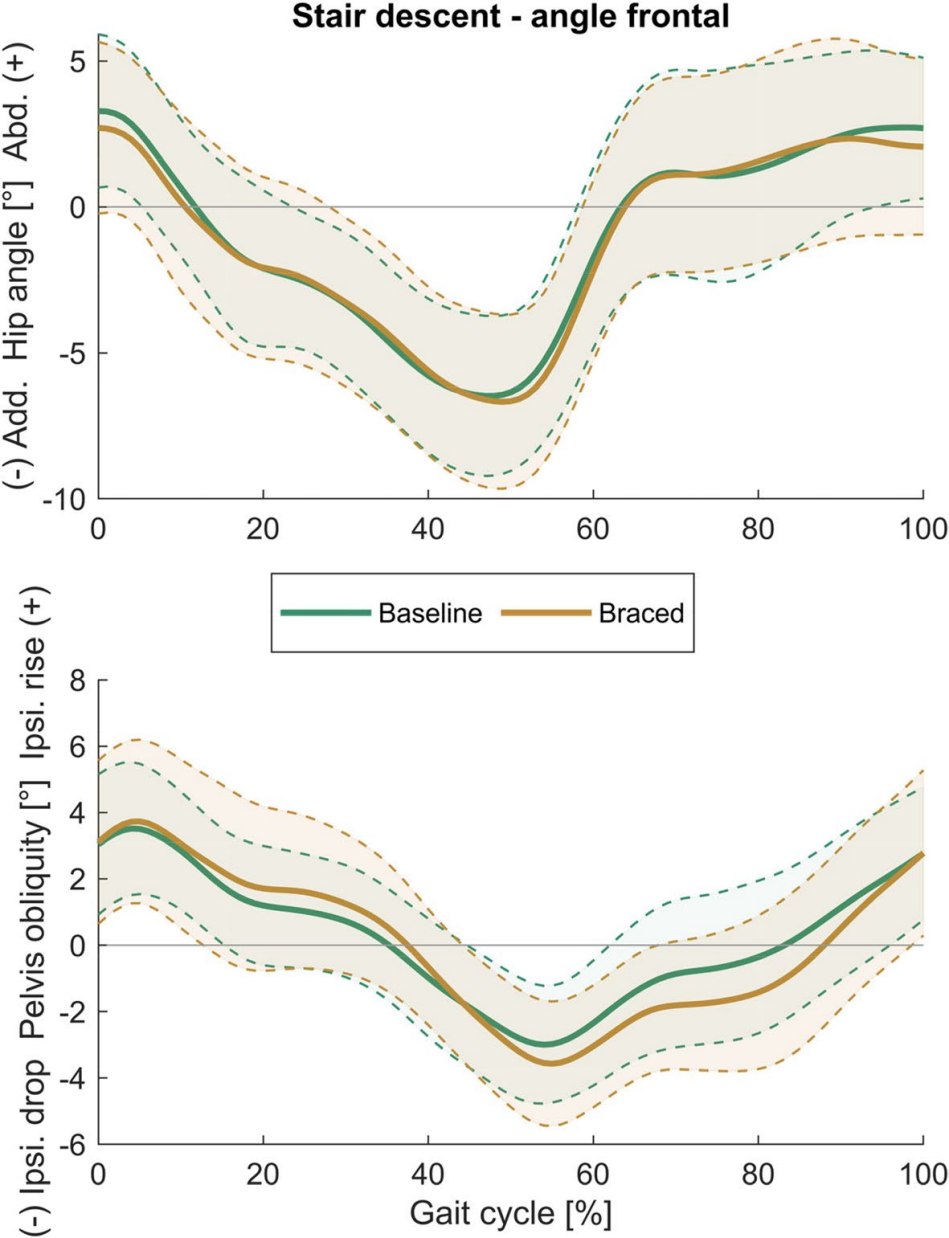
Frontal plane hip and pelvis angles during stair descent. (Mean ± SD)

Tables 1 and 2 present descriptive data for all biomechanical parameters, along with the results of the analyses for stair ascent and descent, respectively. Time curves of the hip and pelvis angles in the sagittal and frontal plane are shown in Figs. 3, 4, 5 and 6. Time curves for the hip angle in the transverse plane and 3D hip moments are included in the Supplementary Material (Figures S1- S4).

**Table 1:** Spatiotemporal, hip, pelvis, and trunk parameters during stair ascent at baseline and after one week of brace application (braced)

Parameter during stair ascent	Baseline	Braced	Mean difference	t-test/WCX
	**mean (SD)**	**mean (SD)**	**(95% CI)**	***p* (|d|)**
Peak hip extension [°]	− 9.50 (4.57)	− 12.77 (4.97)	**3.27 (1.24 5.30)**	**0.003 (0.68)**
Peak hip flexion [°]	48.61 (6.80)	43.79 (7.45)	**4.82 (3.47 6.16)**	**< 0.001 (0.66)**
Hip sagittal range of motion [°]	58.11 (5.55)	56.56 (6.02)	1.55 (− 0.02 3.12)	0.053 (0.26)
Peak hip extension moment [Nm/kg]	0.53 (0.38)	0.49 (0.38)	0.04 (− 0.10 0.18)	0.559 (0.10)
Peak hip flexion moment [Nm/kg]	− 1.13 (0.41)	− 1.07 (0.49)	− 0.06 (− 0.22 0.09)	0.143 (0.14)
Peak backward trunk lean [°]	− 3.07 (14.27)	− 1.89 (12.86)	− 1.18 (− 3.00 0.65)	0.194 (0.08)
Peak forward trunk lean [°]	0.79 (14.50)	2.14 (12.93)	− 1.35 (− 3.15 0.45)	0.133 (0.09)
Peak pelvis posterior tilt [°]	9.82 (3.72)	6.86 (3.57)	**2.96 (1.61 4.30)**	**< 0.001 (0.81)**
Peak pelvis anterior tilt [°]	14.71 (3.38)	12.76 (2.83)	**1.95 (0.61 3.29)**	**0.007 (0.62)**
Pelvis sagittal range of motion [°]	4.90 (1.71)	5.90 (2.02)	**− 1.01 (− 1.84 − 0.18)**	**0.020 (0.53)**
Peak hip adduction [°]	− 9.03 (4.02)	− 7.43 (3.08)	**− 1.60 (− 2.70 − 0.50)**	**0.007 (0.42)**
Peak hip abduction [°]	3.38 (2.83)	4.41 (3.00)	− 1.03 (− 2.18 0.12)	0.076 (0.35)
Hip frontal range of motion [°]	12.41 (3.54)	11.84 (3.15)	0.57 (− 0.54 1.67)	0.294 (0.17)
Peak hip adduction moment [Nm/kg]	− 0.48 (0.21)	− 0.53 (0.22)	0.04 (− 0.06 0.15)	0.729 (0.20)
Peak hip abduction moment [Nm/kg]	0.19 (0.14)	0.18 (0.13)	0.01 (− 0.03 0.05)	0.927 (0.09)
Peak contralateral trunk lean [°]	− 2.59 (1.07)	− 2.59 (1.49)	0.00 (− 0.75 0.75)	0.997 (0.00)
Peak ipsilateral trunk lean [°]	2.26 (2.31)	2.77 (1.93)	− 0.51 (− 1.38 0.36)	0.236 (0.24)
Peak ipsilateral pelvis drop [°]	− 5.12 (2.03)	− 6.43 (1.83)	**1.31 (0.56 2.06)**	**0.002 (0.68)**
Peak ipsilateral pelvis rise [°]	3.91 (1.75)	4.70 (2.27)	**− 0.79 (− 1.54 − 0.04)**	**0.040 (0.37)**
Pelvis frontal range of motion [°]	9.03 (2.70)	11.13 (3.10)	**− 2.10 (− 3.17 − 1.03)**	**0.001 (0.72)**
Peak hip int. rotation [°]	− 4.00 (10.76)	− 0.33 (10.07)	**− 3.67 (− 7.13 − 0.20)**	**0.039 (0.35)**
Peak hip ext. rotation [°]	6.40 (11.07)	9.44 (10.61)	− 3.03 (− 7.17 1.10)	0.330 (0.27)
Hip transverse range of motion [°]	10.40 (1.87)	9.77 (3.67)	0.63 (− 1.01 2.28)	0.432 (0.21)
Peak hip internal rotation moment [Nm/kg]	− 0.13 (0.07)	− 0.14 (0.06)	0.01 (− 0.02 0.04)	0.447 (0.16)
Peak hip external rotation moment [Nm/kg]	0.17 (0.06)	0.17 (0.07)	0.00 (− 0.02 0.03)	0.875 (0.03)
Pelvis transverse range of motion [°]	6.78 (2.10)	6.95 (2.76)	− 0.18 (− 1.51 1.16)	0.786 (0.07)
Stance phase duration [s]	0.79 (0.14)	0.75 (0.12)	**0.05 (0.02 0.08)**	**0.005 (0.34)**
Stride duration [s]	1.23 (0.19)	1.17 (0.17)	**0.06 (0.02 0.11)**	**0.010 (0.34)**
Mean horizontal CoM velocity [m/s]	0.47 (0.07)	0.49 (0.08)	**− 0.02 (− 0.04 0.00)**	**0.021 (0.27)**
Mean vertical CoM velocity [m/s]	0.30 (0.04)	0.31 (0.05)	**− 0.01 (− 0.02 0.00)**	**0.021 (0.25)**

External hip moments

Level of significance < 0.05; significant results in bold

*SD* standard deviation, *95% CI* 95% confidence interval, *WCX* Wilcoxon test, *CoM* centre of mass

**Table 2:** Spatiotemporal, hip, pelvis, and trunk parameters during stair descent at baseline and after one week of brace application (braced)

Parameter during stair descent	Baseline	Braced	Mean difference	t-test/WCX
	**mean (SD)**	**mean (SD)**	**(95% CI)**	**p (|d|)**
Peak hip extension [°]	− 6.13 (5.74)	− 8.73 (7.12)	2.60 (0.71 4.49)	**0.009 (0.38)**
Peak hip flexion [°]	23.92 (9.14)	19.08 (9.39)	4.85 (3.30 6.39)	**< 0.001 (0.52)**
Hip sagittal range of motion [°]	30.06 (5.72)	27.81 (5.92)	2.25 (0.74 3.76)	**0.006 (0.39)**
Peak hip extension moment [Nm/kg]	1.52 (0.34)	1.68 (0.36)	− 0.15 (− 0.26 − 0.05)	**0.005 (0.44)**
Peak hip flexion moment [Nm/kg]	− 0.14 (0.19)	− 0.10 (0.12)	−0.04 (− 0.12 0.03)	0.985 (0.26)
Peak backward trunk lean [°]	0.71 (3.71)	− 0.38 (4.44)	1.08 (− 0.46 2.63)	0.159 (0.26)
Peak forward trunk lean [°]	5.48 (3.72)	4.12 (4.49)	1.35 (− 0.23 2.93)	0.089 (0.32)
Peak pelvis posterior tilt [°]	0.76 (4.50)	− 1.29 (4.49)	2.05 (0.99 3.10)	**0.001 (0.46)**
Peak pelvis anterior tilt [°]	4.85 (4.48)	3.43 (4.02)	1.43 (0.41 2.44)	**0.008 (0.33)**
Pelvis sagittal range of motion [°]	4.10 (1.34)	4.72 (1.77)	− 0.62 (− 1.32 0.08)	0.078 (0.39)
Peak hip adduction [°]	− 7.02 (2.68)	− 7.35 (2.95)	0.33 (− 0.54 1.19)	0.441 (0.11)
Peak hip abduction [°]	4.51 (2.80)	4.20 (3.04)	0.32 (− 0.63 1.26)	0.493 (0.11)
Hip frontal range of motion [°]	11.54 (3.85)	11.55 (3.91)	− 0.01 (− 1.23 1.21)	0.475 (0.00)
Peak hip adduction moment [Nm/kg]	− 0.35 (0.23)	− 0.51 (0.48)	0.16 (−0.03 0.35)	0.083 (0.39)
Peak hip abduction moment [Nm/kg]	0.56 (0.28)	0.50 (0.34)	0.06 (− 0.08 0.20)	0.360 (0.20)
Peak contralateral trunk lean [°]	− 1.71 (1.25)	− 1.41 (1.49)	− 0.30 (− 0.94 0.35)	0.349 (0.21)
Peak ipsilateral trunk lean [°]	1.43 (1.48)	1.47 (1.71)	− 0.04 (− 0.72 0.64)	0.902 (0.03)
Peak ipsilateral pelvis drop [°]	− 3.27 (1.83)	− 3.86 (1.92)	0.59 (− 0.26 1.44)	0.164 (0.31)
Peak ipsilateral pelvis rise [°]	3.79 (1.98)	4.00 (2.49)	− 0.21 (− 1.07 0.65)	0.617 (0.09)
Pelvis frontal range of motion [°]	7.06 (2.24)	7.86 (3.00)	− 0.80 (− 2.07 0.47)	0.203 (0.30)
Peak hip int. rotation [°]	− 3.22 (9.56)	0.56 (10.14)	− 3.79 (− 7.63 0.05)	0.053 (0.38)
Peak hip ext. rotation [°]	10.13 (10.51)	11.30 (9.78)	− 1.17 (− 4.73 2.39)	0.499 (0.11)
Hip transverse range of motion [°]	13.36 (4.60)	10.74 (3.22)	2.62 (0.74 4.49)	**0.009 (0.64)**
Peak hip internal rotation moment [Nm/kg]	− 0.19 (0.12)	− 0.17 (0.14)	− 0.02 (− 0.08 0.04)	0.216 (0.15)
Peak hip external rotation moment [Nm/kg]	0.23 (0.10)	0.29 (0.12)	− 0.06 (− 0.12 0.00)	**0.037 (0.56)**
Pelvis transverse range of motion [°]	8.93 (3.07)	6.74 (2.54)	2.19 (1.42 2.96)	**< 0.001 (0.74)**
Stance phase duration [s]	0.70 (0.13)	0.66 (0.10)	0.04 (0.01 0.06)	**0.013 (0.29)**
Stride duration [s]	1.09 (0.19)	1.03 (0.14)	0.06 (0.01 0.10)	**0.020 (0.31)**
Mean horizontal CoM velocity [m/s]	0.53 (0.09)	0.56 (0.09)	− 0.03 (− 0.05 − 0.01)	**0.016 (0.30)**
Mean vertical CoM velocity [m/s]	0.32 (0.06)	0.34 (0.06)	− 0.02 (− 0.03 0.00)	**0.024 (0.29)**

External hip moments

Level of significance < 0.05; significant results in bold

*SD* standard deviation, *95% CI* 95% confidence interval, *WCX* Wilcoxon test, *CoM* centre of mass

### Effects of hip bracing on pain perception

Participants wore the brace for an average of 10.1 (± 3.6) hours each day. The average pain score during stair walking decreased from 24.9 (± 17.6) at baseline to 17.9 (± 19.7) in the intervention period (MD = − 7.0; *p* = 0.008). Increases in mean pain scores of 10.3 (± 7.5) mm or 39.3 (± 8.0) % were observed for three participants, while decreases in mean pain scores of 10.1 (± 6.6) mm, or 49 (± 24.8) % were observed for the remaining 17 participants.

### Effects of hip bracing on movement velocity

During stair ascent and descent, bracing increased the horizontal (ascent: MD = − 0.02 m/s, *p* = 0.021; descent: MD = − 0.03 m/s, *p* = 0.016) and vertical CoM velocity (ascent: MD = − 0.01 m/s, *p* = 0.021; descent: MD = − 0.02 m/s, *p* = 0.024) and reduced the stance phase (ascent: MD = 0.05 s, *p* = 0.005; descent: MD = 0.04 s, *p* = 0.013) and stride duration (ascent: MD = 0.06 s, *p* = 0.010; descent: MD = 0.06 s, *p* = 0.020).

### Effects of hip bracing on hip biomechanics

During stair ascent, bracing increased the peak hip extension angle (MD = 3.27°, *p* = 0.003) and decreased the peak hip flexion angle (MD = 4.82°, *p* < 0.001), peak hip adduction angle (MD = − 1.60°, *p* = 0.007) and peak internal rotation angle (MD = − 3.67°, *p* = 0.039).

During stair descent, bracing decreased the peak hip flexion angle (MD = 4.85°, *p* < 0.001) and sagittal and transverse hip RoM (sagittal: MD = 2.25°, *p* = 0.006; transverse: MD = 2.62°, *p* = 0.009), and increased the peak hip extension angle (MD = 2.60°, *p* = 0.009), peak hip extension moment (MD = − 0.15 Nm/kg, *p* = 0.005) and the peak external rotation moment (MD = − 0.06 Nm/kg, *p* = 0.037).

### Effects of hip bracing on pelvis and trunk motion

During stair ascent, bracing increased peak pelvis posterior tilt angle (MD = − 2.96°, *p* < 0.001) and decreased peak pelvis anterior tilt angle (MD = 1.95°, *p* = 0.007), resulting in an increased sagittal pelvis RoM (MD = − 1.01°, *p* = 0.020). Moreover, bracing increased peak ipsilateral pelvis rise and drop (rise: MD = − 0.79°, *p* = 0.040; drop: MD = 1.31°, *p* = 0.002), resulting in a significant increase of frontal pelvis RoM (MD = − 2.10°, *p* = 0.001).

During stair descent, bracing increased the peak posterior pelvis tilt (MD = 2.05°, *p* = 0.001) and decreased the peak anterior pelvis tilt (MD = 1.43°, *p* = 0.008) and the transverse pelvis RoM (MD = 2.19°, *p* < 0.001).

Bracing did not influence trunk motion during stair ascent or descent.

### Correlation of changes in stair walking biomechanics and pain perception

Tables 3 and 4 present the correlations between pain perception and biomechanical parameters for stair ascent and descent, respectively. Scatter plots can be found in the Supplementary Material (Figures S5 & S6).

**Table 3:** Correlation between change in perceived pain during stair walking and change in biomechanical **stair ascent** parameters

Parameter during stair ascent	Pearson’s r	95% CI	p
Pelvis peak ipsilateral rise [°]	0.39	− 0.06 0.71	0.090
Pelvis frontal range of motion [°]	0.38	− 0.08 0.70	0.100
Pelvis sagittal range of motion [°]	0.36	− 0.09 0.70	0.114
Hip peak int. rotation [°]	− 0.33	− 0.67 0.13	0.155
Hip peak extension angle [°]	0.29	− 0.17 0.65	0.211
Hip peak adduction angle [°]	0.22	− 0.25 0.60	0.348
Pelvis peak ipsilateral drop [°]	− 0.15	− 0.56 0.31	0.527
Pelvis peak posterior tilt [°]	− 0.12	− 0.54 0.34	0.610
Pelvis peak anterior tilt [°]	0.10	− 0.36 0.52	0.663
Mean horizontal CoM velocity [m/s]	0.06	− 0.39 0.49	0.795
Hip peak flexion angle [°]	− 0.03	− 0.47 0.42	0.902
Stance phase duration [s]	0.02	− 0.43 0.46	0942
Mean vertical CoM velocity [m/s]	− 0.01	− 0.45 0.44	0.980
Stride duration [s]	0.00	− 0.44 0.44	0.992

*95% CI* 95% confidence interval, *CoM* centre of mass

**Table 4:** Correlation between change in perceived pain during stair walking and change in biomechanical **stair descent** parameters

Parameter during stair descent	Pearson’s r	95% CI	p
Hip transverse range of motion [°]	− 0.41	− 0.72 0.04	0.071
Hip peak extension [°]	0.31	− 0.15 0.66	0.177
Hip sagittal range of motion [°]	− 0.27	− 0.64 0.20	0.249
Pelvis transverse range of motion [°]	− 0.25	− 0.62 0.22	0.297
Mean horizontal CoM velocity [m/s]	0.24	− 0.22 0.62	0.311
Mean vertical CoM velocity [m/s]	0.24	− 0.23 0.62	0.299
Pelvis peak posterior tilt [°]	− 0.19	− 0.59 0.27	0.415
Stride duration [s]	− 0.15	− 0.56 0.31	0.515
Stance phase duration [s]	− 0.14	− 0.55 0.33	0.567
Hip peak ext. extension moment [Nm/kg]	0.13	− 0.33 0.54	0.580
Hip peak flexion [°]	0.12	− 0.34 0.53	0.616
Pelvis peak anterior tilt [°]	0.07	− 0.38 0.50	0.764
Hip peak ext. external rotation moment [Nm/kg]	0.03	− 0.42 0.46	0.910

*95% CI* 95% confidence interval, *CoM* centre of mass

None of the biomechanical factors affected by brace application showed a significant correlation with the change in pain perception. However, four parameters during stair ascent (Table 3, Figure S5) and two during stair descent (Table 4, Figure S6) showed moderate correlation coefficients. Decreases in pain score were moderately correlated with decreased pelvis peak ipsilateral rise angle (*r* = 0.39), decreased pelvis frontal (*r* = 0.38) and sagittal RoM (*r* = 0.36), as well as decreased peak hip internal rotation angle (*r* = − 0.33) during stair ascent.

For stair descent, increased hip peak extension angle (*r* = 0.31) and increased hip transverse range of motion (r = − 0.41) were moderately correlated with decreases in pain score.

## Discussion

The present study aimed to assess the effects of one week of hip bracing on hip, pelvis, and trunk biomechanics, as well as pain perception, during stair walking in individuals with mild-to-moderate HOA. We found that bracing reduced hip pain during stair walking by approximately 28%. Nérot and Nicholls [9] reported a reduction in pain of 18 (± 18) mm for nine subjects and an increase in pain of 33 (± 15) mm in five subjects during level walking with bracing. Compared to this study, the present reduction of 10.1 mm in 18 subjects and increase in pain of 10.3 mm in 3 subjects is smaller yet more consistent across subjects and additionally obtained during a more strenuous movement task. Tubach et al. [33] propose a minimal clinically important difference (MCID) of 15.3 mm, or 32%. In this perspective the relevance of the changes observed in pain score is questionable. However, Bird & Dickson showed that the MCID depends on the initial pain level, with lower initial pain levels leading to a lower MCID [34]. In the study from Tubach et al. [33] individuals had an initial pain score of 56.7 (± 16.5), which was therefore much higher than in the present study, with a mean baseline VAS of 24.9 (± 17.6). Hence, an MCID of 15.3 mm might overestimate the change in VAS needed in the present cohort. Moreover, Dekker [35] stresses that the MCID is highly subjective, and that the individual patient must decide which improvement is important enough to undergo a treatment. On an individual level, 14 of the 21 subjects in this study reported changes in VAS > 32% (range 33.3 to 84.7). Therefore, despite the observed absolute change in mean VAS being relatively low, we assume that bracing on an individual level led to a clinically meaningful change in their pain perception for most subjects.

Decreased stair walking velocity has been often observed in individuals with HOA [10, 23, 24]. The biomechanical analysis showed that, in line with our hypothesis, bracing increased movement velocity during both stair ascent and descent. The observed decrease in hip pain likely enabled this increase in stair walking velocity. Thus, bracing was able to reverse the detrimental effect of HOA on stair walking velocity. As during steady-state stair walking only minor modifications of step length are possible due to the dimensions of the step, the increased velocity was caused by an increase in cadence, as indicated by lower stride durations. Potential implications of the increase in cadence on dynamic balance and risk of falling should be addressed in future research.

Stair descent requires substantial control of the downward movement of the CoM. Increased muscular co-contraction to stiffen the limb in single support has been described as a strategy to reduce vertical CoM velocity and, as a result, reduce the muscular demand for CoM deceleration and the joint contact forces at IC [36]. Therefore, increasing the vertical CoM velocity with bracing likely required greater muscle activity to decelerate the CoM and potentially increased the hip contact force. Thereby, it has been found that the activity of the periarticular musculature is important for shock absorption [37], and greater GMed activation was associated with reduced impact forces during a step-down task in individuals with HOA [38]. Therefore, alterations in GMed activity caused by the friction massage of the brace might mediate the effect of increased contact force due to increased CoM velocity and decreased contact force due to periarticular muscle activity. Additionally, the GMed has been found to contribute to the forward acceleration of the CoM during stair descent [39] and thus enhanced GMed activation could also have caused the observed increase in anteroposterior CoM velocity. However, since muscle activity and joint loading were not captured during this study, further investigation is warranted.

The increased movement velocity likely contributed to the observed increase in peak hip extension and external rotation moments during stair descent, as gait velocity correlates with hip joint kinematics and dynamics [40, 41]. Reduced peak hip extension and external rotation moments have previously been described in individuals with HOA during stair descent [10, 21]; bracing thus counteracted these effects of HOA on stair descent dynamics. However, larger hip extension moments require larger activity of the hip flexors, which are weakened in individuals with mild-to-moderate HOA [42]. Thus, increasing velocity and peak hip extension moments during stair descent might promote muscle fatigue, but simultaneously exercise weakened hip flexor muscles. Future studies should address the long-term effect of hip brace use on the force capacity of the hip muscles.

Although the brace applied in the current study did not aim to alter the femur position mechanically, in line with our hypothesis, it reduced peak hip internal rotation during stair ascent by 3.7° and reduced the transverse hip RoM during stair descent by 2.6°. Thereby, reduced hip internal rotation angles during stair ascent were moderately correlated to reduced pain during stair walking. In contrast, a moderate correlation was found between decreased transverse hip RoM during stair descent and increased hip pain. Other braces intending to mechanically externally rotate the hip joint resulted in higher reductions of peak hip internal rotation angles of 6.7° during level walking in individuals with HOA [9] and of 4.2° and 6.4° during stair ascent and descent, respectively, in individuals with femoroacetabular impingement [43]. However, in those studies, no effect on pain perception was found [43], or mixed results were reported [9]. Preventing RoM endpoints from being reached is thought to reduce hip pain [44, 45] and Altman et al. [12] included pain on hip internal rotation as an important aspect for diagnosis of HOA. Peak hip internal rotation was observed at 20% of the gait cycle combined with hip adduction and flexion and in a period of high joint loading as the entire bodyweight must be lifted against gravity by one leg. In contrast, the reduction in transverse hip RoM correlated to an increase in pain during stair descent where peak hip internal rotation occurs during the swing phase and thus a period of marginal loading. Hence, the reduction of peak internal rotation in phases of high joint loading might be an important aspect for pain reduction. Therefore, future studies need to clarify the effect of hip internal rotation on pain in individuals with HOA. As results differ between movement tasks, analyses should focus on the situation in which peak internal rotation occurs, e.g. in combination with hip adduction and flexion, or the simultaneous loading condition.

In contrast to our hypothesis, limited frontal hip mobility in individuals with HOA was aggravated by brace use, with reductions in peak hip adduction angle of 1.6° during stair ascent. Similar results have previously been reported, with reductions of 1.9° during level walking [9] and approximately 3° in stair walking [43]. Limiting hip adduction has been identified as a strategy to increase mediolateral stability and reduce hip contact forces [46]; however, this approach also decreases the demand on the hip abductor muscles, potentially leading to muscle weakness in the long term [11]. Despite the reduction in peak adduction, we did not observe a decrease in the external adduction moment, which is closely linked to hip contact forces [46], either due to the simultaneous increase in movement velocity or a change in frontal pelvis mobility.

It remains unclear whether the reductions in peak hip internal rotation and adduction angles are due to the passive resistance of the hip brace as the integrated hinge joint only provides one degree of freedom in the sagittal plane, or whether it results from an altered movement strategy and muscle activation pattern. As the brace did not affect peak hip adduction angle during stair descent and level walking [7], passive resistance seems less likely. However, the observed peak hip adduction angles were greatest during stair ascent. Since the brace applies a friction massage to the GMed above the greater trochanter, altered activity of the GMed could have caused the observed changes in hip adduction. The GMed not only counteracts the hip adduction moment, but also contributes to external rotation of the hip joint in phases of low hip flexion and to internal rotation of the hip joint in phases of high hip flexion [47]. Therefore, altered activity of the GMed could also have impacted peak hip rotation.

Furthermore, brace application led to a less anteriorly tilted pelvis and shifted the sagittal peak hip angles towards decreased flexion and increased extension. As the hip joint angle is calculated by the motion of the femur relative to the pelvis, this shift in hip angles is likely caused by the more upright pelvis position, which results in a more extended position of the hip joint without altering the femur’s position in global space. A typical posture in individuals with HOA is characterized by lumbar lordosis and increased anterior pelvis tilt [48], which is a compensatory mechanism for reduced hip extension ability [49]. Hip bracing thus counteracted the postural adaptation described for individuals with unilateral [49] and bilateral moderate-to-severe HOA [50, 51].

The observed reduction in peak flexion of 4.8 is comparable to the 5.3° previously reported in individuals with femoroacetabular impingement during stair walking [43]. Limiting peak hip flexion during stair walking may be beneficial, as greater hip flexion angles during level walking [52] and stair walking [53] have been associated with faster disease progression.

The observed increase in peak hip extension likely causes and increase in hip contact force, as increased hip extension at terminal stance has been associated with increased and redirected contact forces in previous studies [46]. For KOA, it has been shown that excessive knee loading drives KOA progression [54] while the results for HOA are equivocal. Modifications in gait patterns of individuals with HOA have often been linked to reduced joint loading and pain avoidance [11], and high peak contact hip stress has been found to increase the risk of hip replacement surgery [55]. However, gait interventions that increase hip contact forces have been associated with reduced hip pain [56]. Likewise, in our study, the observed increases in hip moments and hip peak extension might have led to increased hip contact forces but were accompanied by a reduction in hip pain. Thereby, increased peak hip extension was correlated to decreased pain perception during stair descent (Table 4; Figure S6). Therefore, future studies should clarify the impact of hip bracing on hip contact forces, as an optimal amount of hip loading may be beneficial in reducing hip pain.

Apart from the hip joint, bracing in our study led to an increase in frontal pelvis motion with increased ipsilateral pelvis rise and drop. Although reduced frontal pelvis RoM has been found during level walking in individuals with various degrees of HOA [49, 57–59], analyses of stair walking did not yield any differences to healthy controls [10, 21]. However, in contrast to level walking, stair ascent requires greater concentric effort from the abductor muscles to elevate the pelvis on the contralateral side to facilitate step clearance of the swinging leg [14]. Increased oblique pelvis motion observed with bracing may result from increased SIJ mobility or as a compensatory motion for reduced hip flexion necessary for step clearance during stair ascent. Furthermore, reducing the contralateral pelvis drop during level walking can increase hip joint contact forces by 11.9% [56], yet, still resulting in decreased hip pain. Peak ipsilateral pelvis rise angle and frontal pelvis RoM showed the strongest correlation with change in pain score in our study (Table 3; Figure S5), and larger increases in pelvis rise and frontal RoM led to larger increases in pain. Thus, the increased frontal pelvis mobility, especially the increased contralateral pelvis drop observed with bracing, seems to be detrimental to the improvements in hip pain. As the GMed is the main stabilizer of the pelvis, these kinematic changes might also originate in altered GMed activity, which should be addressed in the future.

Our study is the first to assess the effects of one week of hip bracing on pain perception and biomechanics during stair walking in individuals with HOA. Using a combination of radiographic and functional HOA assessments allowed us to ensure that participants are representative of the target group for conservative treatment methods, as opposed to other studies that included participants with end-stage HOA [9]. Furthermore, the higher wear comfort of elastic hip braces, as applied in the current study, likely increases therapy adherence compared to braces constructed to unload the hip joint, which are often reported to be uncomfortable [43, 60, 61].

Furthermore, stair walking biomechanics were assessed at the midpoint of a six-step instrumented staircase and are therefore representative of a steady-state stair walking motion, as opposed to transition steps between level and stair walking, often reported in other studies [10, 22].

Besides these strengths of our study, some limitations should also be considered. The evaluation of hip and pelvis biomechanics using optical motion capture necessitates the placement of markers on anatomical landmarks. In the braced condition, it was not feasible to attach markers directly to the skin above the anterior and posterior superior iliac spines. Consequently, markers for the anterior spine were placed on the hip belt, while markers for the posterior spine were affixed through the mesh fabric of the pelvis belt. Applying markers to clothing rather than to the skin increases the risk of relative motion between the anatomical landmark and the marker [62]. Alternative methods, such as clusters, are often reported in knee brace analyses [63, 64] but are not applicable for the hip joint as the brace entirely covers the pelvis, and the adjoining segments (thighs and torso) exhibit substantial portions of wobbling mass. However, as the pelvis belt of the brace was fitted very tightly and stair walking movements are relatively slow in comparison to running or jumping, relative movement between the pelvis and the brace is likely to be minimal, although it cannot be entirely ruled out.

The observed changes in hip kinematics induced by bracing were 2.6° to 4.8° in the sagittal plane, 1.6° in the frontal plane, and 3.7° in the transverse plane. Small changes always raise questions about their clinical relevance. However, bracing also induced reductions in pain perception, and some parameters were moderately correlated with this change. Thus, while the absolute changes were small, they might still have affected pain perception. Overall, interpreting transverse plane biomechanics requires caution, as marker-based motion capturing is susceptible to errors in this plane [65]. For hip biomechanics, accurately estimating the hip joint centre is crucial [66]. Therefore, the equations of Harrington et al. [29] were used for hip joint centre estimation in our study, as they are the most accurate regression method [67].

To eliminate the effects of bilateral involvement, we excluded individuals with K-L grade 3 or 4 contralateral radiographic HOA, as well as those with limitations in passive joint RoM. The thresholds for an unrestricted passive RoM were derived from clinical practice and the expertise of one of the authors. However, as there are no scientific reference values for passive hip RoM in this age group, limitations in contralateral hip RoM cannot be definitively ruled out.

Furthermore, it was not possible to blind participants to the bracing condition, which raised the risk of placebo effects, especially in subjective parameters such as pain perception.

Lastly, the effect of bracing was evaluated after only one week of wearing the brace. As bracing is likely to be used over a more extended period in the conservative treatment of HOA, the long-term effects must be evaluated before recommendations for or against its use can be made.

## Conclusion(s)

We examined the effect of hip bracing on hip pain and biomechanics of the hip, pelvis, and trunk during steady-state stair walking in individuals with functional mild-to-moderate HOA. Bracing of only one week significantly reduced hip pain and reversed some of the HOA-induced alterations in stair walking biomechanics by increasing movement velocity, peak hip extension angle, and peak hip extension and external rotation moments, while decreasing anterior pelvis tilt. Thereby, reductions in hip internal rotation in a period of high joint loading during stair ascent and increases in hip extension during stair descent were moderately correlated with reductions in pain score. These findings can be used to further understand and investigate the underlying mechanisms of pain reduction and functional improvement observed with bracing or other conservative treatment methods. In contrast to previous studies, the brace used in this study primarily comprised elastic components which target the manipulation of soft tissue around the pelvis, hip and ISJ and several of the observed kinematic and dynamic changes are indicative of a change in hip abductor muscle activity. Therefore, future studies should evaluate the effect of hip bracing on muscle activation. Moreover, increased hip extension, higher movement velocity and higher CoM velocity could potentially increase loading of the hip joint but occurred simultaneously to the decrease in hip pain. Further analyses on the impact of hip bracing on joint loading and its relation to hip pain are required especially with regard to a more long-lasting use of the hip brace.

All in all, our results indicate that hip bracing is effective in reducing hip pain, enhancing posture and improving hip function during the demanding task of stair walking. Therefore, bracing should be considered in the conservative treatment of mild-to-moderate HOA as a low-cost option that allows patients to self-manage their therapy.

## Supplementary Information

Supplementary Material 1.

## Data Availability

The datasets used during the current study are available from the corresponding author upon reasonable request.

## Abbreviations

BMI: Body mass index
CoM: Centre of mass
GMed: Gluteus medius
GRF: Ground reaction force
HOA: Hip osteoarthritis
IC: Initial contact
K-L: Kellgren-Lawrence
KOA: Knee osteoarthritis
RoM: Range of motion
MCID: Minimal clinically important difference
MD: Mean difference
SIJ: Sacroiliac joint
SD: Standard deviation
VAS: Visual analogue scale
WCX: Wilcoxon test
95% CI: 95% Confidence interval
