# Supplementary material for: Effect of hip bracing on stair walking biomechanics and pain in patients with mild-to-moderate hip osteoarthritis: an intervention study
Source: BMC Musculoskelet Disord. 2026 Feb 11;27:174. doi: 10.1186/s12891-026-09587-2 (PMC12930933; doi:10.1186/s12891-026-09587-2)
Supplement: Supplementary file 1 — Supplementary Material 1. [file 12891_2026_9587_MOESM1_ESM.pdf]

Supplementary Material

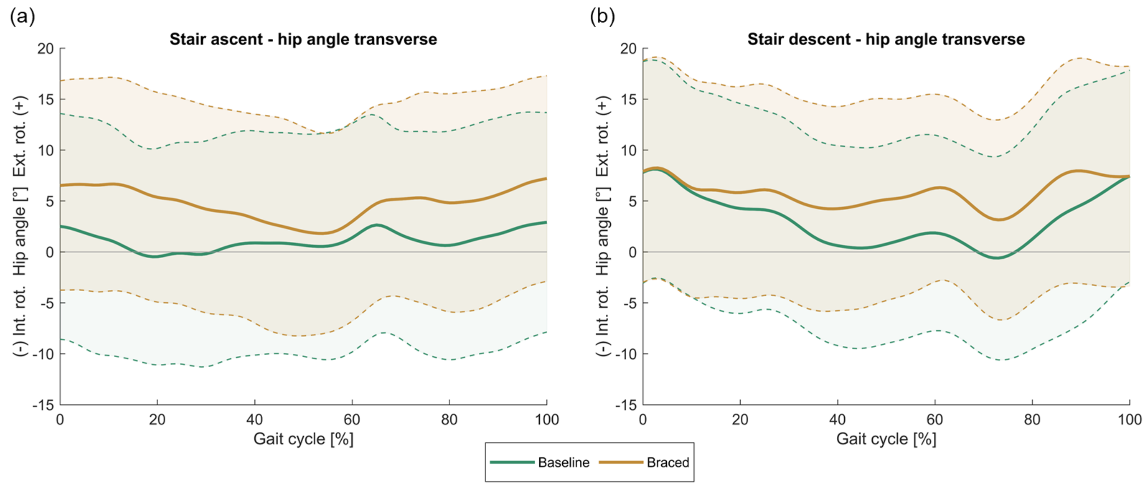

Figure S1: Transverse plane hip angle (Mean ± SD) across the gait cycle during (a) stair ascent and (b) stair descent.

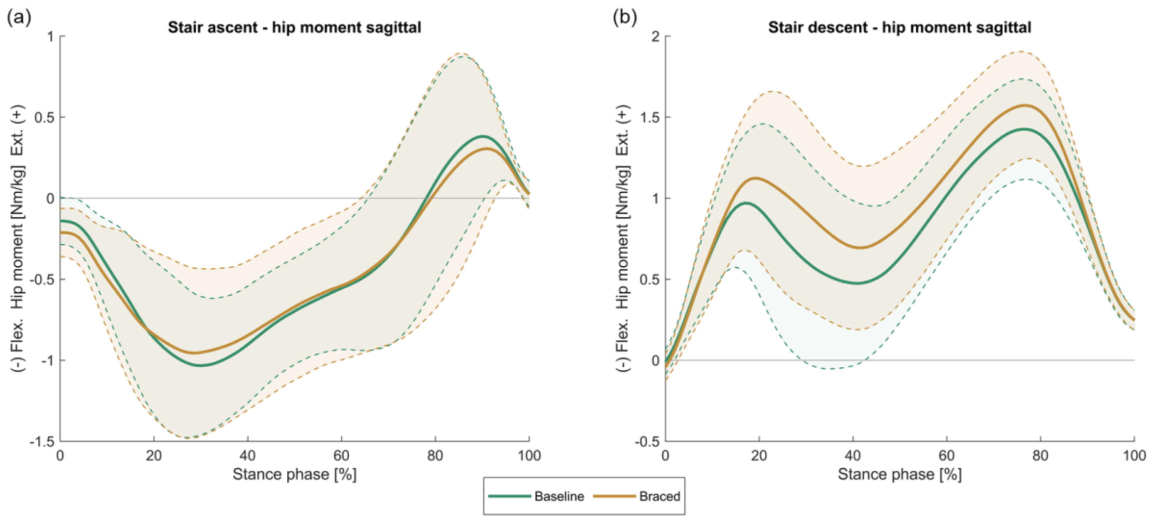

Figure S2: Sagittal plane external hip moments [Nm/kg] (Mean ± SD) across the stance phase during (a) stair ascent and (b) stair descent.

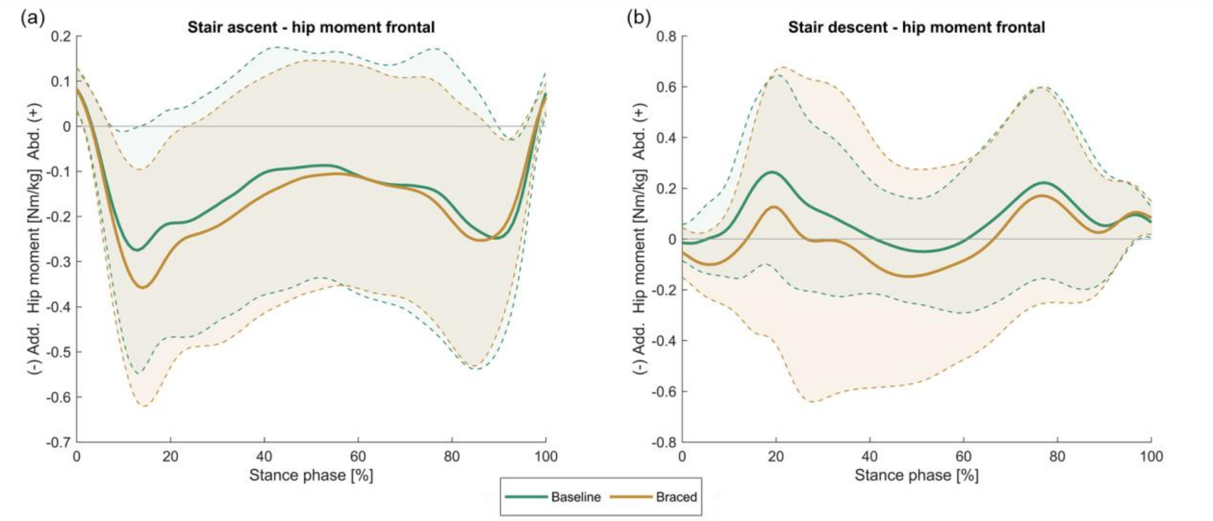

Figure S3: Frontal plane external hip moments [Nm/kg] (Mean ± SD) across the stance phase during (a) stair ascent and (b) stair descent.

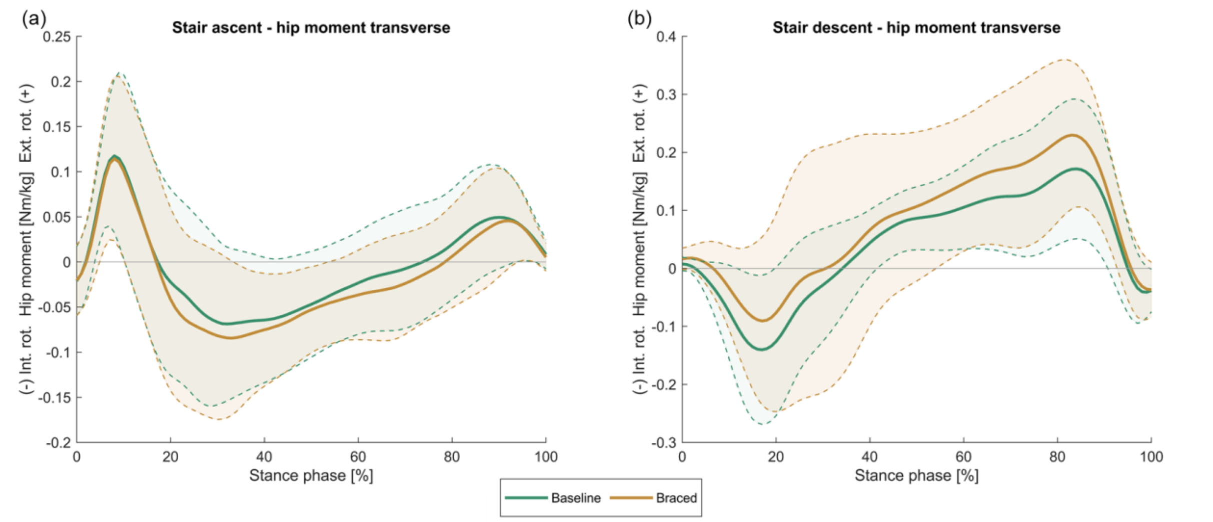

Figure S4: Transverse plane external hip moments [Nm/kg] (Mean ± SD) across the stance phase during (a) stair ascent and (b) stair descent.

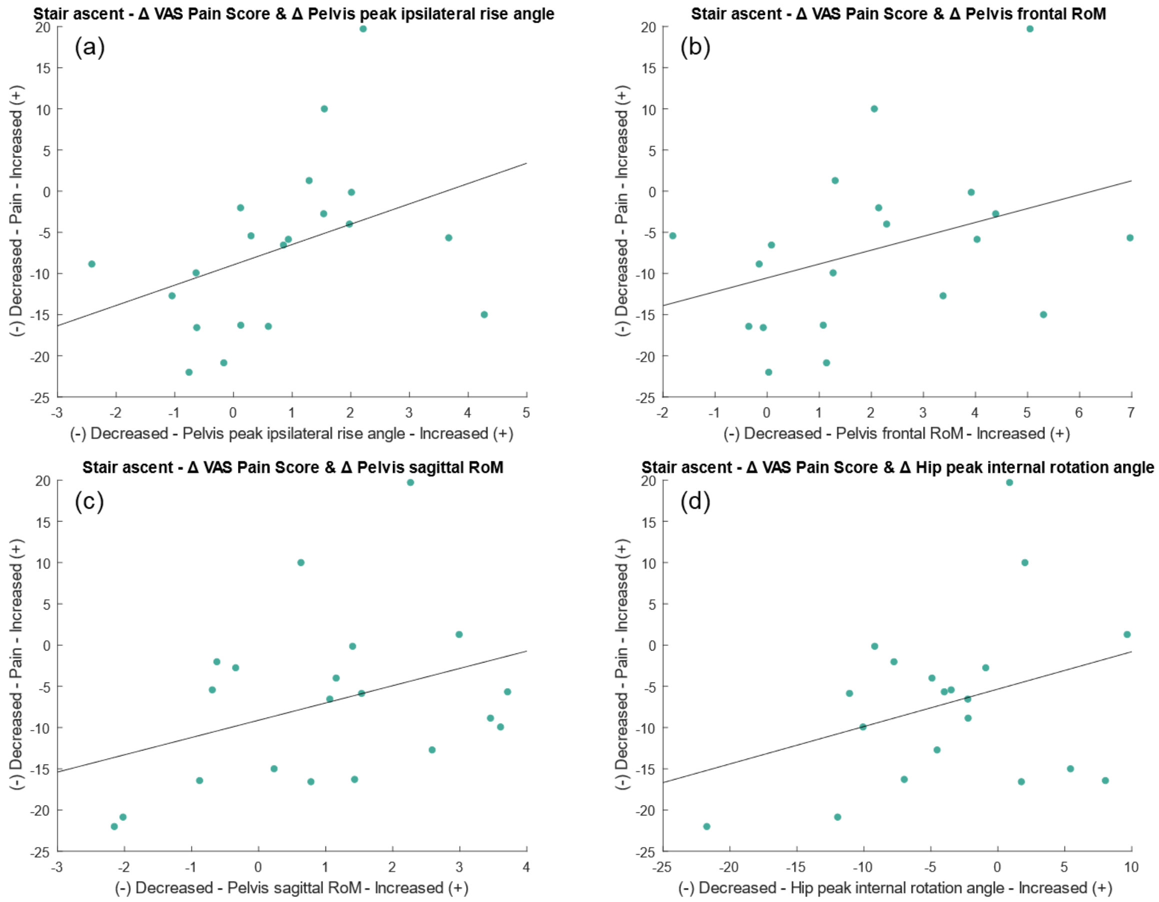

Figure S5: Scatter plots of Δ VAS pain score versus Δ (a) pelvis peak ipsilateral rise angle, (b) pelvis frontal RoM, (c) pelvis sagittal RoM, (d) hip peak internal rotation angle during stair ascent.

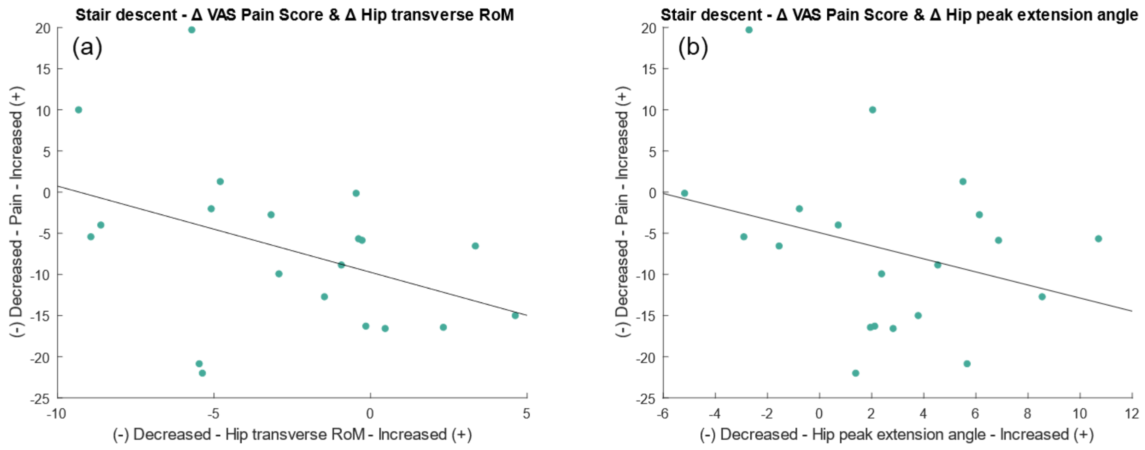

Figure S6: Scatter plots of Δ VAS pain score versus Δ (a) hip transverse RoM and (b) hip peak extension angle during stair descent.
